# Innate Killing of *Leishmania donovani* by Macrophages of the Splenic Marginal Zone Requires IRF-7

**DOI:** 10.1371/journal.ppat.1000813

**Published:** 2010-03-12

**Authors:** Rebecca Phillips, Mattias Svensson, Naveed Aziz, Asher Maroof, Najmeeyah Brown, Lynette Beattie, Nathalie Signoret, Paul M. Kaye

**Affiliations:** 1 Centre for Immunology and Infection, Hull York Medical School and Department of Biology, University of York, York, United Kingdom; 2 Center for Infectious Medicine, Department of Medicine, F59, Karolinska Institutet, Karolinska University Hospital, Huddinge, Stockholm, Sweden; 3 The Technology Facility, Department of Biology, University of York, York, United Kingdom; Imperial College London, United Kingdom

## Abstract

Highly phagocytic macrophages line the marginal zone (MZ) of the spleen and the lymph node subcapsular sinus. Although these macrophages have been attributed with a variety of functions, including the uptake and clearance of blood and lymph-borne pathogens, little is known about the effector mechanisms they employ after pathogen uptake. Here, we have combined gene expression profiling and RNAi using a stromal macrophage cell line with in situ analysis of the leishmanicidal activity of marginal zone macrophages (MZM) and marginal metallophilic macrophages (MMM) in wild type and gene targeted mice. Our data demonstrate a critical role for interferon regulatory factor-7 (IRF-7) in regulating the killing of intracellular *Leishmania donovani* by these specialised splenic macrophage sub-populations. This study, therefore, identifies a new role for IRF-7 as a regulator of innate microbicidal activity against this, and perhaps other, non-viral intracellular pathogens. This study also highlights the importance of selecting appropriate macrophage populations when studying pathogen interactions with this functionally diverse lineage of cells.

## Author Summary

Macrophages are phagocytic cells that play a dual role in infection. They can kill ingested micro-organisms and thus help eliminate the threat from infection, but some pathogens have adapted to survive within macrophages and use the intracellular niche they provide as a means of immune evasion. Although it has long been recognised that macrophages in different tissues look and behave differently, this heterogeneity is rarely taken into account when examining macrophage-pathogen interactions. By comparing gene expression profiles of different types of macrophages, we show here the diversity of the macrophage response to *Leishmania donovani* infection. In one cell line that resembles the macrophages of the spleen that are targets of *L. donovani* infection, we identified a gene expression signature more similar to that expected for a viral infection, and by using RNAi, identified that the transcription factor IRF-7 played an essential role in the selective capacity of these macrophages to kill this intracellular parasite. The importance of this pathway of host resistance was directly confirmed by examining the response of splenic macrophages in *Irf-7*-deficient mice. This comparative approach has, therefore, identified a new molecular pathway involved in the innate tissue-specific control of *L. donovani* infection.

## Introduction

Mononuclear phagocytes are widely distributed in all tissues, and provide a broad range of homeostatic and immune functions during development and throughout adult life. Nevertheless, the heterogeneity of mature tissue macrophages represents one of the most striking, yet under-studied, features of mononuclear cell differentiation. Expression of a range of transcription factors and cellular receptors has helped define membership of the mononuclear phagocyte system [1],[2],[3], and whereas some tissue macrophages have a capacity for local self-renewal, others are derived from blood-borne monocytes undergoing tissue-specific differentiation [reviewed in [4]]. In lymphoid tissues, macrophage heterogeneity is most strikingly evident. For example in mice, readily distinguishable populations of macrophages are found in the splenic MZ, red pulp, B cell follicles and white pulp [5]. Moreover, within the MZ, SIGNR-1^+^MARCO^+^ MZM occupy the outer rim adjacent to the red pulp, and CD169^hi^ MMM border the lymphocyte-rich white pulp [5],[6],[7],[8],[9],[10]. Mice lacking various transcription factors (e.g. relβ and NFκB2), TNF superfamily cytokines (e.g. LTα), and chemokines (e.g. CCL19/21ser) exhibit steady-state defects in MZ macrophage differentiation and/or positioning [11],[12], illustrating the complexity behind this micro-anatomical organisation.

MZM and MMM are well-placed in the marginal sinus to encounter blood borne antigens and pathogens. MZM are avidly phagocytic, are well-characterised as facilitating clearance of *Streptococcus pneumoniae* from the blood stream [13],[14],[15] and are involved in the initiation of Type I T-independent immune responses [16]. MMM, whilst also phagocytic [17] are better recognised for playing a role in antigen transport into B cell follicles [4] and are also known to be robust producers of IFN-α following infection with viral pathogens [18]. MMM, and to a lesser extent MZM, also express CD169, a sialoadhesin first described on bone marrow ‘stromal’ macrophages involved in the support of erythropoiesis [19] and these cells may provide this or other stromal functions in the spleen. Similarly, in lymph nodes, subcapsular sinus macrophages mark a point of entry for viruses and bacteria entering via the afferent lymphatics and are intimately involved in the initiation of antibody responses [20],[21],[22]. Thus, various specialised macrophages populations are ideally situated within secondary lymphoid tissues to first encounter pathogens that enter the blood and lymphatics.

Nevertheless, despite their importance in providing a ‘gatekeeper’ function, little is known about how these tissue resident macrophages subsequently deal with the pathogens they encounter. Direct study of MZM and MMM has been problematic and only rarely reported [6], largely because of the inherent technical difficulties in isolating these scarce and fragile cells for functional analysis in vitro. Hence, our understanding of the effector functions of MZM and MMM is largely extrapolated from that of other diverse macrophage populations, such as those derived from the peritoneal cavity or grown from bone marrow precursors under the influence of CSF-1. In this study, therefore, we set out to directly identify the mechanism by which splenic MZM and MMM are able to kill intracellular *Leishmania* parasites. We first identified a stromal macrophage cell line (14M1.4; [23]) that displayed similar innate capacity to kill *L. donovani* as observed in MZM and MMM. By gene expression profiling, we showed that *Leishmania* infection stimulated expression of the transcription factor *Irf-7*, as well as redistribution of cellular IRF-7 from MyD88^+^ endosomes to the *Leishmania*-containing phagosome. Initial control of intracellular *Leishmania* by 14M1.4 cells involved NO and was minimally affected by silencing of *Irf-7*. However, subsequent leishmanicidal activity strictly required *Irf-7* and was NO-independent. Furthermore, *Irf-7* loss-of-function could not be overcome by exogenous IFN-α, suggesting a downstream effector mechanism uncoupled from *Irf-7*-dependent amplification of IFN-α. By immunohistochemistry of spleens from infected mice, we showed that MZM and MMM also responded to infection with heightened expression and phagosomal recruitment of IRF-7. Finally, we showed that MZM and, to a lesser extent, MMM from *Irf-7*-deficient mice were unable to kill intracellular *Leishmania*. By focusing on these tissue-resident stromal macrophages, we have thus identified a new role for IRF-7 as a regulator of macrophage anti-leishmanial activity, and demonstrated that this transcription factor plays a role in innate effector responses to a broader range of intra-phagosomal pathogens than previously recognised.

## Results

### IFN-γ-independent killing of *Leishmania* by stromal macrophages

**Figure 1:**
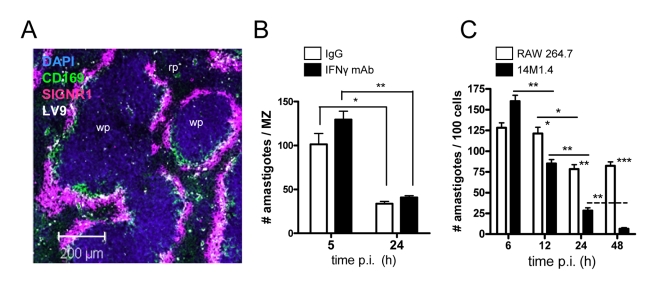
Splenic marginal zone macrophages and marginal metallophilic macrophages control *L. donovani* by an IFN-γ independent mechanism. (**A**) *L. donovani* amastigotes are found predominantly in MMM and MZM after intravenous injection (purple, SIGNR1; green CD169; white, *L. donovani*; blue DAPI). (**B**) C57BL/6 mice were treated with 0.5mg XMG 1.6 anti-IFN-γ IgG (black bars) or control IgG (open bars) and 2h later infected with *L. donovani*. At 5h and 24h p.i., the number of parasites per MZ area was determined. (**C**) 14M1.4 cells (black bars) and RAW 264.7 cells (open bars) were infected with *L. donovani* (MOI 10∶1) and at indicated times p.i., parasite numbers per 100 macrophages were determined by fluorescence microscopy. *, *p*<0.05, **, *p*<0.01 between bars indicated as indicated. Scale bar; 200µm.

Previous studies in BALB/c mice showed that after i.v. injection, *L. donovani* amastigotes are rapidly and selectively taken up by MZM and MMM, and that over the following 24h, parasite load in the MZ was significantly reduced [17]. We first confirmed this selectivity of uptake in the MZ of C57BL/6 mice, observing greater than 90% of amastigotes in the MZ (within cells staining either for CD169 or SIGNR1) and few parasites in either the deep white pulp or in the red pulp (**Figure 1A**). To confirm that C57BL/6 mice also expressed rapid leishmanicidal activity and to determine whether this might be mediated by NK cell-derived IFN-γ [24], we treated mice with either control IgG or anti-IFN-γ. Both control and anti-IFN-γ-treated mice reduced parasite load to a similar extent, indicating that this response was largely IFN-γ independent (**Figure 1B**). We then screened a number of macrophage cell lines (RAW264.7, J774.1, 14M1.4) and primary macrophage populations (bone marrow-derived, peritoneal) for their ability to kill *L. donovani* amastigotes in the absence of IFN-γ activation. Of the cells tested, 14M1.4 cells were striking in their ability to clear amastigotes over a 24–48h period. Infected 14M1.4 cells reduced parasite load by ∼50% from 6h–12h after infection and sustained this activity over the subsequent 36h, resulting in almost complete clearance of amastigotes by 48h (**Figure 1C**). In contrast, although RAW264.7 reduced the number of intracellular amastigotes by ∼30–40% from 12h–24h, no further reduction in parasite load was detected after this time. Differences in amastigote numbers were not readily attributable to differential rates of cell division between 14M1.4 cells and other cells tested, with 14M1.4 and RAW264.7 cells showing similar levels of CFSE dilution over 48h (**Supplementary Figure S1A**). Likewise, equivalent levels of CFSE dilution were observed in intracellular amastigotes isolated from both cell populations, indicating that parasite growth rate was similar in these two host cells (**Supplementary Figure S1B**). Thus, these data collectively demonstrated that 14M1.4 cells were intrinsically very efficient at killing intracellular amastigotes.

### Gene expression profiling of stromal macrophages

**Figure 2:**
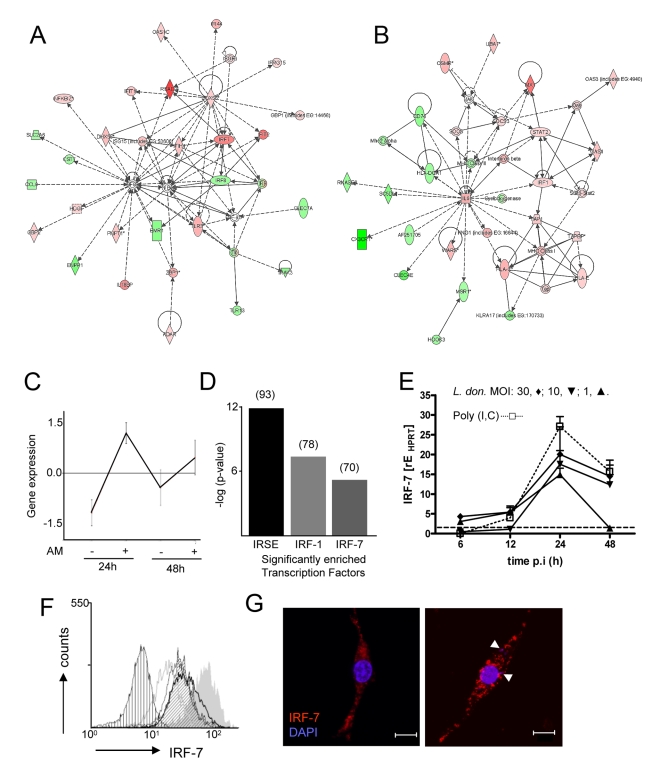
Induction of an interferon-response signature in 14M1.4 cells following *L. donovani* infection. Expression profiling of 14M1.4 cells, with and without infection with *L. donovani* was performed using Affymetrix gene array. (**A and B**) Networks showing up (red) and down (green) regulated genes 48h after *L. donovani* infection were generated using Ingenuity Pathways; (A) ‘antimicrobial response’ and (B) ‘antigen presentation’. (**C**) Differentially expressed genes were clustered using CLICK. 550 genes were significantly induced at both 12h and 48h p.i. (**D**) Transcription factor enrichment analysis using PRIMA identified IRF-7, IRF-1 and ISRE as significantly enriched in the 550 gene cluster shown in (C). Number of genes in each set is indicated in brackets. (**E**) Real-time RT-PCR showing *Irf-7* mRNA accumulation in 14M1.4 following exposure to *L. donovani* at MOI of 30∶1 (diamond), 10∶1 (invert triangle), 1∶1 (triangle) or after exposure to 100µg/ml poly(I:C) (open square) . (**F**) Kinetics of IRF-7 expression on 14M1.4 cells following exposure to *L. donovani* at a MOI of 10∶1 for 6 (open grey line), 12 (open thin black line), 24h (open thick black line) or 48h (angled hatch) or to 100µg/ml poly (I:C) (filled gray). Control untreated cells are also shown (vertical hatch) (**G**) Expression of IRF-7 by confocal microscopy (red, IRF-7; blue, DAPI) in uninfected (left) and *L. donovani*- infected (right) 14M1.4 cells. Amastigotes are indicated by arrowheads. Scale bar: 10µm.

To understand further the mechanism(s) by which 14M1.4 cells killed intracellular amastigotes, we used global gene expression profiling to examine the transcriptional response of 14M1.4 cells to *L. donovani* infection. 977 and 851 genes were found to be differentially expressed in infected vs. control 14M1.4 cells at 12h or 48h p.i. respectively, using a cut-off of a 2-fold change in gene expression (**Supplementary Tables S1 and S2**). These differentially expressed genes were analysed by generating gene interaction networks using Ingenuity Pathways Analysis software. 10 networks of high interconnectivity (≥20 differentially regulated genes) were constructed for genes differentially regulated at 12h p.i. and 12 hubs at 48h p.i (**Supplementary Figure S2 and S3**). In contrast, only 136 and 161 genes were differentially regulated in RAW264.7 cells, at 12h and 48h p.i. respectively (**Supplementary Tables S3 and S4**), and only one hub with high interconnectivity could be identified at each time point (**Supplementary Figure S2 and S3**). In 14M1.4 cells, the highest scoring networks reflected genes involved in interferon signalling (notably *Irf-7*, *Irf-1*, *Stat-2*), cytokines (notably *Il-6*) and cardinal interferon response genes (including *mx-1*, *Rsad* and various MHC and MHC related genes: **Figure 2A and B**). At 48h, 31% of genes (9/29) listed in the Ingenuity Pathways ‘Interferon Signalling’ pathway were represented in the differentially induced gene set, all being upregulated. Within the ‘Antigen Presentation Pathway’, 25% (10/39) were either up- or down-regulated, with a marked differential response between MHCI-and MHCII-associated genes (**Figure 2B**). A range of genes within these networks and associated with pathogen recognition and handling were also down-regulated including *Irf-8*, recently associated with the innate control of intra-phagosomal pathogens [25], *Cx3cr1*, a chemokine receptor involved in the NO-dependent bactericidal activity of macrophages [26], *Clec7a* (Dectin-1) and *Clec4a* (Mincle), both associated with fungal recognition [27],[28] and *Msr1*, the macrophage scavenger receptor and its cytoplasmic partner Hook3 [29],[30]. *Sc5d*, associated with cholesterol synthesis [31] was also down-regulated, in keeping with data indicating that *Leishmania* infection depletes this membrane lipid [32],[33], as was *Emr1* (F4/80; [34]). TGFβ and a network of interconnected genes were also specifically down-regulated at 48h in 14M1.4 cells (**Supplementary Figure S3**).

We next performed clustering using the CLICK algorithm contained in EXPANDER [35] which identified 550 genes in a single cluster (homogeneity value: 0.895) that were increased in their expression level at both 12h and 48h p.i. (**Figure 2C**). GO functional classes representing ‘immune response’ (GO:0006955; 5.45%) and ‘defense response’ (GO: 0006952; 0.54%) were significantly enriched in this cluster. We next used PRIMA software [36] to identify transcription factor sequences enriched amongst these 550 induced genes. By this analysis (**Figure 2D**), we identified *Irf-7* and *Irf-1* and ISRE, as potential regulators of the response of 14M1.4 cells to *L. donovani*. Similar analysis of genes down regulated in 14M1.4 cells at both time points did not reveal any significantly enriched transcription factors (data not shown).

Based on the high levels of expression in 14M1.4 cells, absence of expression of an interferon response signature in RAW264.7 cells, and the known role of *Irf-7* as a central regulator of the Type I interferon response [37],[38], we focused further attention on *Irf-7*. Confirmation of the induction of *Irf-7* following *Leishmania* infection was obtained by quantitative RT-PCR (**Figure 2E**), by intracellular flow cytometry (**Figure 2F**) and by confocal microscopy (**Figure 2G**). Amastigotes induced a dose- and time-dependent accumulation of *Irf-7* mRNA and IRF-7 protein in 14M1.4 cells that was similar in kinetics but of somewhat reduced magnitude than poly(I:C) a known Toll-like receptor (TLR)-3-dependent inducer of *Irf-7*. In contrast, RAW264.7 cells and bone marrow-derived macrophages induced minimal levels of *Irf-7* mRNA accumulation or IRF-7 protein expression (**Supplementary Figure S4**). Together, these data indicated that 14M1.4 stromal macrophages, but not RAW 264.7 macrophages responded to *L. donovani* infection by strong induction of IRF-7.

### IRF-7 is recruited to the *Leishmania* phagosome

**Figure 3:**
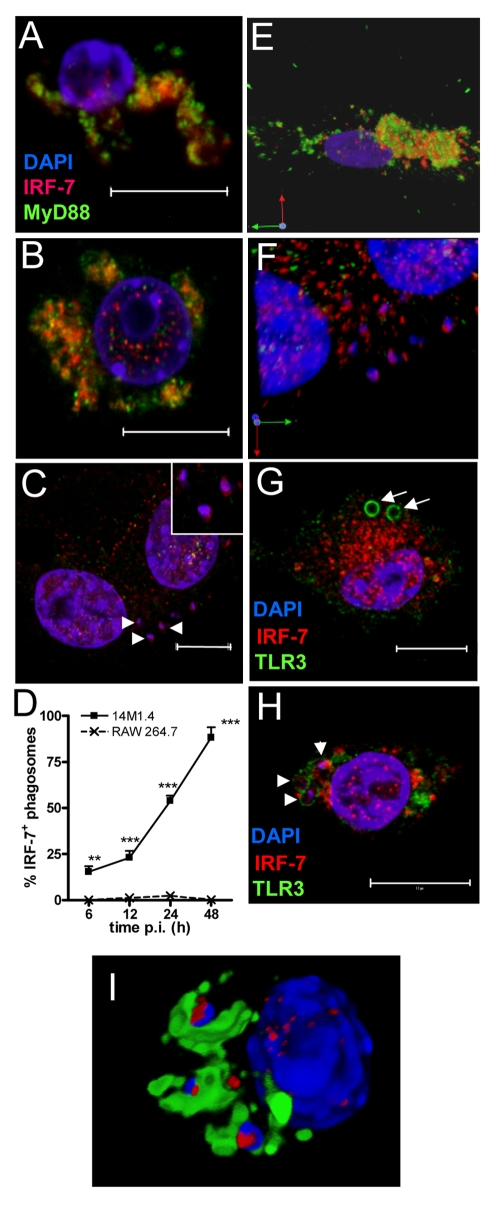
IRF-7 is recruited to *Leishmania* phagosomes. Untreated (**A**), poly(I:C)-treated (**B**) and 48h *L. donovani*-infected (**C**) 14M1.4 cells were stained for IRF-7 (red) and MyD88 (green). Cells were counterstained with DAPI (blue) and analysed by confocal microscopy. Single confocal slices are shown. Amastigotes are indicated with arrowheads and enlarged in inset in (**C**). (**D**) Frequency of IRF-7^+^ phagosomes was scored at time intervals shown in infected 14M1.4 and RAW264.7 cells. (**E, F**) Snap shots from Video S1 and S2, showing IRF-7-MyD88 association in untreated 14M1.4 cells (E) and phagosomal ‘capping’ of IRF-7 in *Leishmania*-infected 14M1.4 cells (F) (red, IRF-7; green, MyD88; blue, DAPI.). (**G**) 14M1.4 cells stimulated with poly (I:C) and allowed to phagocytose latex beads (arrow), stained for IRF-7 (red) and TLR3 (green). (**H**) 14M1.4 cells infected with *L. donovani* and stained for TLR3 (green) and IRF-7 (red). Scale bars; 10µm. (**I**) Snap shot from Video S3, showing IRF-7 and LAMP1 staining in 24h *Leishmania*-infected 14M1.4 cells (red, IRF-7; green, MyD88; blue, DAPI).

In IRF-7/MyD88 co-transfected RAW264.7 and HEK 293 cells and in plasmacytoid DC, IRF-7 and MyD88 co-localise in endosomes [39]. To establish whether such co-localisation also occurred in un-manipulated 14M.1.4 cells and to gain further insight into IRF-7 activation in these cells, we examined the distribution of MyD88 and IRF-7 by confocal microscopy (**Figure 3**). In resting 14M1.4 cells, IRF7 and MyD88 were largely co-localised in small cytoplasmic vesicles, with little evidence of nuclear IRF-7 (**Figure 3A** **; Video S1, shown as a snapshot in** **Figure 3E**). Following poly(I:C) activation, nuclear translocation of IRF-7 occurred, with residual cytoplasmic IRF-7 remaining associated with MyD88 containing vesicles (**Figure 3B**). In sharp contrast, although 14M1.4 cells infected for 48h with *L. donovani* also had nuclear IRF-7, cytoplasmic IRF-7 was now primarily associated with phagosomes containing *Leishmania*, and rarely with MyD88^+^ vesicles (**Figure 3C**). By 48h p.i., almost all *L. donovani* phagosomes were IRF-7^+^ (**Figure 3D**). IRF-7 was almost undetectable in RAW264.7 macrophages, with or without infection, but where low levels of IRF-7 could be seen in rare cells, an association with the *L. donovani* phagosome was not apparent (**Supplementary Figure S4**). Of interest, IRF-7 in infected 14M1.4 cells was almost always localised to one pole of the phagosome (**Figure 3C** **insert; Video S2, shown as a snapshot in** **Figure 3F**). The recruitment and polarisation of IRF-7 in the *Leishmania* phagosome was specific, based on three sets of observations. First, MyD88 was not similarly detected at this location (**Figure 3C and F**). Second, in 14M1.4 macrophages treated with poly(I:C) and then exposed to latex beads for 48h, no phagosomal recruitment of IRF-7 was observed in spite of high levels of induction of IRF-7 (**Figure 3G**). Third, TLR3 accumulating on latex and *Leishmania* phagosomes did not show the polar distribution seen with IRF-7 on *Leishmania* phagosomes (**Figure 3G and H**). With TLR3 used to demarcate the phagosome membrane, IRF-7 appeared to have an intra-phagosomal localisation (**Figure 3H**), an observation that was supported by using LAMP1 as an alternative phagosome membrane marker (**Figure 3I** **and Video S3**). Although some co-localisation with LAMP1 was observed, a significant amount of the observed IRF-7 was clearly intra-phagosomal, either on, within or tightly apposed to the amastigote itself. In summary, these data demonstrate that dissociation of endosomal MyD88 and IRF-7 is followed by nuclear and phagosomal recruitment of IRF-7.

**Figure 4:**
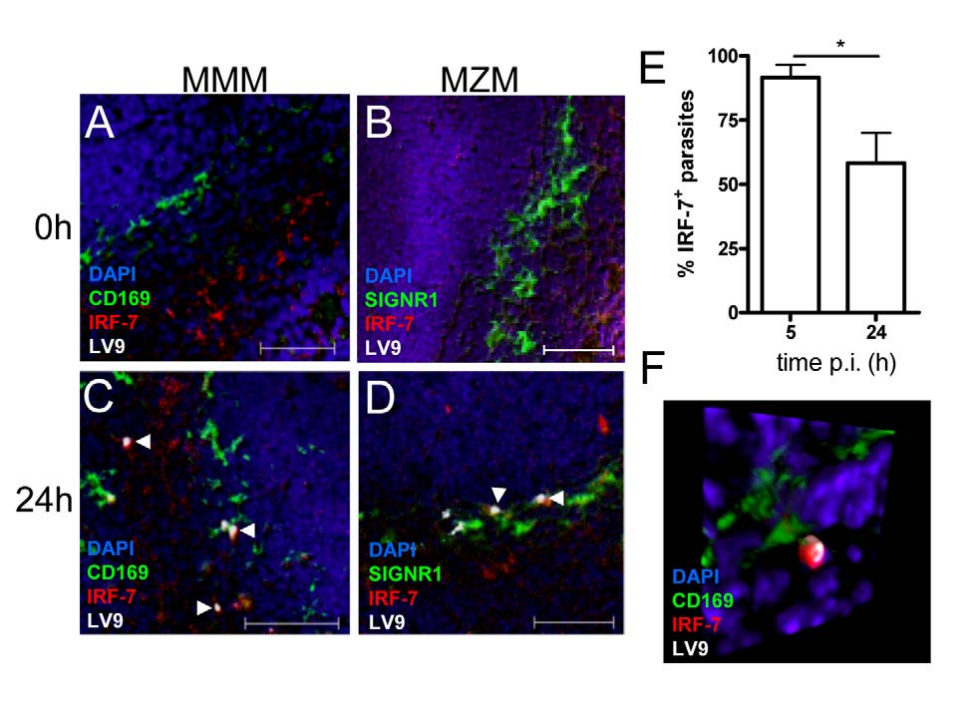
IRF-7 is induced in MZM and MM in situ. (**A–D**) Induction of IRF-7 in MMM and MZM. Naïve (A,B) and C57BL/6 mice infected for 24h (C, D) were stained for CD169 (A, C; green) or SIGNR1 (B, D; green), IRF-7 (red) and *L. donovani* (white). (**E**) The percentage of IRF-7^+^ parasite-containing phagosomes was quantified by counting 100 amastigotes/section (n = 3) and calculating the proportion of parasites with IRF-7 accumulation. *, *p*<0.05 (**F**) Snap shot from Video S4, showing infected MMM with phagosomal localisation of IRF-7 (CD169^+^ green, IRF-7, red; LV9, white; DAPI, blue). Scale bars; 50µm.

To determine whether the IRF-7 response in 14M1.4 cells actually reflected the response of MZM and MMM to *Leishmania* infection in situ, we prepared tissue sections from control and *L. donovani*-infected mice, and analysed IRF-7 expression (**Figure 4**). IRF-7 expression was minimal in MMM (**Figure 4A**) and MZM (**Figure 4B**) of naïve mice, but readily observed at 5h (data not shown) and 24h in MMM (**Figure 4C**) and MZM (**Figure 4D**) of infected C57BL/6 mice. At 5h p.i., ∼90% and at 24h p.i. ∼65% of parasite-containing phagosomes had clearly associated IRF-7 staining (**Figure 4E**), and although it was difficult to always assess the subcellular distribution of IRF-7, a polar distribution, similar to that seen in 14M1.4 cells in vitro, was often observed (**Video S4, shown as a snapshot in** **Figure 4F**). Collectively, these data support the contention that the early leishmanicidal activity of splenic MZ macrophage populations is also accompanied by IRF-7 induction and recruitment of IRF-7 to the amastigote-containing phagosome.

### IRF-7 is required for leishmanicidal activity

**Figure 5:**
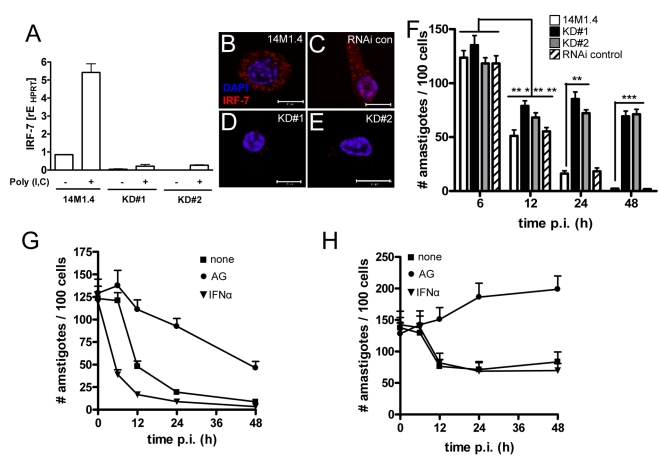
IRF-7-dependent killing of *Leishmania* in 14M1.4 cells. (**A**) Silencing of *Irf-7* in 14M1.4 cells using lentiviral shRNA generated two clones (KD#1 and KD#2) with >95% knockdown in mRNA accumulation after poly(I:C) induction. (**B–E**) IRF-7 expression in untreated wild type 14M1.4 (B), in RNAi-control 14M1.4 cells (C) and in KD#1 (D) and KD#2 (E) clones. (**F**) Wild type 14M1.4 cells (open bars), RNAi control 14M1.4 cells (hatch bars), KD#1 (black bars) and KD#2 (grey bars) cells were infected with *L. donovani* and the parasite load per 100 cells was determined at the times indicated. *, *p*<0.05, **, *p*<0.01, ***, *p*<0.005 comparing 14M1.4 and knock-down clones. (**G and H**) 14M1.4 (G) and KD#1 (H) cells were treated with aminoguanidine (circle) or exogenous IFN-α (invert triangle) or untreated (square) then infected with *L. donovani*. Infection was determined from replicate cultures at times indicated. Scale bars; 10µm.

To directly determine whether IRF-7 induction was involved in regulating the leishmanicidal activity of 14M1.4 cells, we performed functional gene knock down of IRF-7 using RNAi. Knock-down clones were screened by RT-PCR (**Figure 5A**) and immunofluorescence (**Figure 5B–E**) in the presence or absence of poly(I:C), and two independent clones with strong inhibition of *Irf-7* mRNA accumulation were selected for further study (KD#1 and KD#2). Analysis of the fate of *L. donovani* in *Irf-7* knock-down compared to control cells indicated that control of parasite burden was biphasic with respect to the requirement for *Irf-7*. Amastigote numbers were reduced to a similar extent over the first 12h of infection in all cells tested, suggesting an early phase of leishmanicidal activity that was *Irf-7*-independent. In contrast, sustained leishmanicidal activity, measured over 12–48h, was completely abrogated by knock-down of *Irf-7* (**Figure 5F**). Thus, the leishmanicidal response of 14M1.4 cells consisted of an immediate *Irf-7*-independent component and a late-acting *Irf-7*-dependent component ultimately responsible for amastigote clearance.

### Involvement of NO and IFN-α in *Irf-7*-dependent killing

IRF-7 plays a central role in the amplification of the Type I IFN response [37] and NO has been reported to have a major role as a leishmanicidal effector in macrophages exposed to either Type I IFNs [40] or IFN-γ [41]. We therefore determined whether NO and IFN-α were involved in the killing of *Leishmania* by 14M1.4 cells. Although only minimal amounts of NO could be detected in the supernatants from infected 14M1.4 cells (**Supplementary Figure S5**), treatment of 14M1.4 cells with the Nos2 inhibitor aminoguanidine (AMG) blocked their ability to rapidly kill *L. donovani* as measured over the first 12h p.i. (**Figure 5G**). Likewise, AMG also blocked the early leishmanicidal activity of *Irf-7*-deficient 14M1.4 cells (**Figure 5H**). In contrast, AMG had a minimal effect on the late phase of leishmanicidal activity in 14M1.4 cells (with parasite burden continuing to decrease steadily from 12–48h) or in *Irf-7*-deficient 14M1.4 cells (with amastigote numbers remaining at similar levels or increasing slightly from 12–48h; **Figure 5G, H**). Thus, whereas NO played a role in immediate parasite killing, it had limited involvement in the subsequent *Irf-7*-dependent phase of leishmanicidal activity.

Next, we exposed wild type and knock down 14M1.4 cells to exogenous IFN-α. At early time points (<12h), exogenous IFN-α significantly enhanced the rate at which wild type, but not *Irf-7*-deficient, 14M1.4 cells killed *L. donovani* (**Figure 5G, H**), suggesting that the early augmentation of effector function mediated by exogenous IFN-α required the presence of *Irf-7*. Importantly, however, at later time points (12–48h) exogenous IFN-α was unable to overcome the loss-of-function associated with *Irf-7*-deficiency (**Figure 5H**). Thus, leishmanicidal activity in 14M1.4 macrophages involves i) an early NO-dependent and *Irf-7*-independent component, which can nevertheless be augmented by IFN-α in an *Irf-7*-dependent manner and ii) a late-acting NO-independent, *Irf-7*-dependent component which can not be compensated for by exogenous IFN-α.

### Killing of Leishmania in the MZ requires IRF-7

**Figure 6:**
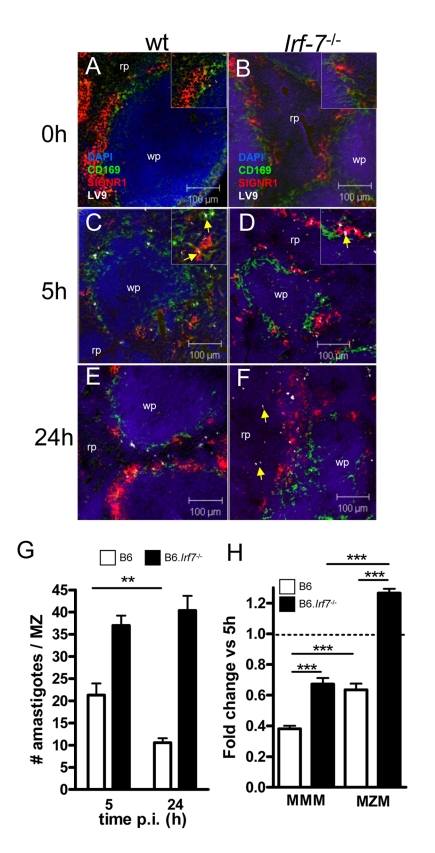
*Irf-7^−/−^* MZM and MMM are defective in anti-leishmanial activity. (**A–F**) Parasite localisation in wild type and *Irf-7^−/−^* mice. Spleens from naïve C57BL/6 (A) and *Irf-7^−/−^* (B) mice and C57BL/6 (C, E) and *Irf-7^−/−^* (D, F) mice infected with *L. donovani* for 5h or 24h, were stained for CD169 (green), SIGNR1 (red), *Leishmania* (white) and counterstained with DAPI (blue). Higher magnification inserts show localization of amastigotes (arrowed). (**G**) Parasite load in the MZ of C57BL/6 mice (open bars) and B6.*Irf-7* ^−/−^ mice (black bars) was determined by counting the number of parasites per MZ area *, *p*<0.05, **, *p*<0.01. (**H**) The number of amastigotes in CD169^+^ MMM and SIGNR1^+^ MZM of B6 (open bars) and *Irf-7^−/−^* (black bars) mice was determined at 5h and 24h (n = 64 MZ profiles, from 4 mice per time point per strain). Data are presented as fold change at 24h. ***, p<0.001. Scale bars; 100µm.

The existence of an *Irf-7*-dependent leishmanicidal pathway in 14M1.4 cells led us to investigate whether innate killing of *Leishmania* in the marginal zone was similarly controlled by IRF-7. In contrast to what has been observed with other interferon regulatory factor-deficient mice, no obvious structural differences were observed in the organisation and cellular content of the marginal zone in *Irf-7* ^−/−^ mice before or after infection (**Figure 6A–F**). However, in contrast to B6 mice, B6.*Irf-7* ^−/−^ mice failed to eliminate intracellular amastigotes over the first 24h of infection (**Figure 6G**). To determine whether *Irf-7*-dependent killing was a property of both MMM and MZM, we separately scored the change in amastigote number within these two populations (**Figure 6H**). In B6 mice, MMM and MZM both significantly reduced their parasite load from 5h to 24h (by 62% and 37% respectively; p<0.001). In *Irf-7* ^−/−^ mice, although MMM were still able to reduce amastigote load by ∼33% (P<0.001), killing activity was clearly reduced in comparison to MMM in wild type mice (p<0.001). For MZM, the impact of *Irf-7*-deficiency was more striking, with *Irf-7* ^−/−^ MZM failing to display any killing activity. Thus, IRF-7 played an essential role in regulating the innate ability of splenic tissue macrophages to kill *L. donovani* in vivo. In the course of these studies, we also noted that in B6.*Irf-7* ^−/−^ , but not in B6 mice, amastigotes could be found more readily within red pulp macrophages (**Figure 6E and F**), suggesting that the barrier function of the marginal zone that prevents early amastigote dissemination may also be impaired by *Irf-7* deficiency.

## Discussion

MZM and MMM have previously been shown to be involved in the uptake of various blood borne pathogens, and in this way provide an important barrier to systemic infection [7],[10],[13],[14],[15],[17],[18]. Although macrophages in the marginal zone have been shown to be capable of IFN-α production [9],[18], their broader effector function has been largely inferred from the in vitro study of other macrophage populations. Here, using a comparative approach involving direct in vitro and in vivo analysis of microbicidal activity, we provide evidence that IRF-7 plays a central role in regulating the ability of MZM and MMM to control the intra-phagosomal pathogen, *L. donovani*. Although IRF-7 has long been acknowledged to orchestrate anti-viral immune responses, a role in macrophage effector responses to non-viral pathogens has not been previously described.

IRF-7 has been shown to co-localise with MyD88 in endosomes of IRF-7 and MyD88 transfected cell lines [39], an observation we confirmed here using non-transfected 14M1.4 cells. Our studies extend these observations by showing that upon *Leishmania* infection, the spatiotemporal link between MyD88 and IRF-7 was lost. Whereas MyD88 remained associated with small cytoplasmic vesicles, IRF-7 was selectively recruited to *Leishmania*-containing phagosomes, both in vitro in 14M1.4 cells and in vivo in infected MZM and MMM. This recruitment of IRF-7 was specific and not observed with latex bead phagosomes, even in cells also containing *Leishmania* or in cells highly activated for IRF-7 expression using poly(I:C). It remains to be formally demonstrated whether this change in IRF-7 localisation reflects i) dissociation of IRF-7 from existing complexes with MyD88 and subsequent re-location to the phagosome, or ii) whether such IRF-7 undergoes nuclear translocation, with only de novo synthesised IRF-7 being recruited to phagosomes. Based on the short half life of IRF-7 [42] and the kinetics of mRNA accumulation described here, we currently favour the latter possibility. Interestingly, the absence of detectable MyD88 on the *Leishmania* phagosome, along with the absence of phagosomal TLR 9 expression (Phillips et al, unpublished observations) suggests that MyD88-dependent phagosomal signaling may not be a significant feature of *Leishmania* infection in these cells, at least at the time points studied. Similarly, it remains to be determined whether phagosomal IRF-7 plays any functional role in the IRF-7-dependent leishmanicidal activity observed here. On the one hand, phagosomal recruitment of IRF-7 might lead to IRF-7 forming part of a signalosome complex at the phagosome membrane, as has been described for phagosome-recruited AP-1 [43]. On the other hand, our confocal analysis suggests that the bulk of phagosomal IRF7 is intra-phagosomal (as defined by LAMP1) and likely to be associated with the amastigote itself. This observation is strikingly similar to that made by Antoine and colleagues who demonstrated acquisition of phagosomal MHCII (and not LAMP1) by *L. amazonensis* amastigotes [44] and suggests that the amastigote might act as a ‘sink’ that accumulates phagosome-associated IRF-7. These two possibilities are not mutually exclusive, and we do indeed see low levels of IRF-7 co-localized with LAMP1 apparently in the phagosome membrane. Nevertheless, given that the amastigotes of *L. donovani* also have tight attachment sites to the phagosomal membrane [45]; and Phillips and Kaye, unpublished), future studies employing immunogold EM, cell fractionation and biochemical approaches will be required to determine the exact localization of IRF-7 in the *L. donovani* phagosome and whether such IRF-7 plays a functional role.

The mechanism of killing we observe in 14M1.4 cells, in MZM and, to a lesser extent, MMM appears distinct from that previously reported for *Leishmania* studied in other macrophage populations. Innate IFN-γ-independent control over *L. donovani* in hepatic and splenic macrophages is a function often attributed to *Slc11a1* [46]. However, this phagosomal iron transporter exerts leishmanistatic control over amastigote growth and, in any case, 14M1.4 cells carry the mutant allele of *Slc11a1*. Similarly, expression profiling suggests that a number of other recently identified anti-microbial pathways are down-regulated in 14M1.4 cells after *Leishmania* infection. Although a role for NO in IFN-γ-independent killing of *Leishmania* has also been established [41], our data indicate that the major phase of *Irf-7*-dependent killing occurs in a largely NO-independent manner. That previous studies have failed to identify this pathway may reflect the stromal origin of 14M1.4 cells and their closer relationship to resident tissue macrophages such as MZM and MMM. For example, RAW264.7 cells, a commonly used cell line in the study of *Leishmania*-macrophage interactions, responds minimally to infection with IRF-7 induction, and hence this pathway would not be evident in studies using this host-pathogen combination. Indeed, our data emphasize the importance of studying appropriate tissue macrophages when aiming to identify physiologically relevant effector mechanisms. Importantly, our data do not argue against a role for IFN-α in the regulation of *Irf-7* [42]. IFN-α could be produced either through an autocrine pathway (as with 14M1.4 cells in vitro) or through a combination of autocrine and paracrine pathways, as is more likely to affect MZM and MMM in vivo. Indeed, plasmacytoid DC (pDC), a major source of IFN-α are readily activated after *Leishmania* infection [[47] and Sanos and Kaye, unpublished). Rather, our finding that exogenous IFN-α could not compensate for *Irf-7* loss-of-function provides evidence for an alternative downstream effector mechanism that cannot be amplified by IFN-α alone. Further studies to identify potential phagosomal effectors regulated by *Irf-7* are currently in progress.

Our studies were not designed to directly address the question of recognition of *L. donovani* during the process of early phagocytic uptake. Nevertheless, recent work has suggested that TLR9 is involved in the recognition of *L. infantum* and *L. major* promastigote-derived genomic DNA by CD11c^+^ cDC and by pDC [48]. In these published studies, however, no attempt was made to determine the immediate consequences of this recognition for the subsequent control of *Leishmania* survival. Using RAW264.7 macrophages, Descoteaux and colleagues suggested a role for both TLR2 and TLR3 in the phagocytic uptake of *L. donovani* promastigotes and a role for TLR3 in the leishmanicidal activity of the IFN-gamma-primed macrophages. However, as reported here, RAW264.7 cells do not recapitulate the IRF-7 response observed in 14M1.4 cells, MZM and MMM and these cells show only limited leishmanicidal activity in the absence of IFN-γ activation. Nevertheless, TLR-3 was induced in *L. donovani*-infected, as well as poly (I:C) treated 14M1.4 cells, but unlike IRF-7, TLR-3 appeared to be promiscuously recruited to phagosome membranes, at least after IRF-7 activation.

CD8^+^ T cell responses are of notable importance for protection against *L. donovani*, and vaccines which target CD8^+^ T cells are particularly effective against this infection in experimental models [49],[50]. Although the role of tissue resident macrophages as antigen presenting cells has recently returned to the fore, with a variety of studies demonstrating antigen capture and presentation to B cells [13], the importance of these cells for the induction of T cell immunity remains controversial [10]. Early studies suggested that innate Type I IFN responses were associated with an increased capacity for cross-priming [51],[52] and might therefore favour the induction of CD8^+^ T cell responses to intracellular pathogens. However, whilst a recent systems biology analysis of the response to Yellow Fever vaccine has confirmed the induction of a Type I IFN gene signature centred on *IRF-7*, this signature was not directly predictive of the magnitude of the CD8^+^ T cell response [53]. Previous studies of MHC gene expression in *Leishmania*-infected macrophages have reported inhibition of basal and IFN-γ-induced MHCI and MHCII gene expression in BALB/c derived peritoneal macrophages [54]. However, in the presence of the strong *Irf-7*-mediated response associated with infection of 14M1.4 cells, we observed a reciprocal regulation of genes related to MHCI- and MHCII-restricted antigen presentation, with elevated levels of transcripts for MHCI, TAP1 and tapasin. A similar role for IRF-7 in regulating cross-presentation has also recently been confirmed following adenoviral gene delivery of *Irf-7* into peritoneal macrophages [55]. Together, these data suggest that further examination of the role of stromal macrophages, as represented by 14M1.4 cells and macrophages of the splenic marginal zone, in CD8^+^ T cell priming is now warranted.

In conclusion, we have shown that IRF-7 is indispensable for effective control of *L. donovani* amastigotes in stromal macrophages *in vitro* and *in vivo*. Our results suggest that IRF-7 may play a similarly important role in the containment of other intracellular pathogens.

## Materials and Methods

### Animals and parasites

C57BL/6 mice (CRUK, Margate, UK) were used at 6–10 weeks of age and housed under specific-pathogen free conditions. B6.*Irf-7^−/−^* mice were obtained from the RIKEN BioResource Center (Ibaraki, Japan) with kind permission of T. Taniguchi, University of Tokoyo, Japan. Amastigotes of the Ethiopian LV9 strain of *L. donovani* were maintained by passage in B6.*RAG1^−/−^* mice. Mice were infected with 2–5×10^7^ amastigotes intravenously in 100µl RPMI 1640 (Gibco, Paisley, UK) and killed by cervical dislocation as required.

### Ethics statement

All experiments were approved by the University of York Animal Procedures and Ethics Committee and performed under UK Home Office license (‘Immunity and Immunopathology of Leishmaniasis’ Ref # PPL 60/3708).

### *In vitro* infection of cell lines

RAW 264.7 macrophages (American Type Culture Collection, Rockville, MD) and 14M1.4 stromal macrophages ([23]; a gift from D. Zipori, Weizmann Institute of Science, Rehovot, Israel) were maintained in complete Dulbecco's modified Eagle's medium (DMEM; Gibco) containing 5% FCS supplemented with 1 mM sodium pyruvate, 2mM l-glutamine, 0.05 mM 2-mercaptoethanol, 100U/ml penicillin and 100µg/ml streptomycin (all Gibco, Paisley, UK). 14M1.4 cells express low levels of SIGNR1 but undetectable levels of MARCO and CD169 (data not shown). 14M1.4 cells deficient in *Irf-7* were generated by transfection with lentiviral particles encoding shRNA for *Irf-7* mRNA (SHVRS-NM_016850, Sigma-Aldrich, St. Louis, MO). Transfected cells were positively selected in complete media containing 5µg/ml puromycin (Sigma-Aldrich, St Louis, MO) and clones were generated by limited dilution. *Irf-7* mRNA knock-down was screened by RT-PCR and immuno-staining. Before use in infection experiments, cells were collected by scraping with cell scraper (BD Falcon) and 5×10^5^ cells in complete DMEM were added to 24 well plates (Corning Inc. Corning, NY) containing sterilised 13mm diameter coverslips (VWR International, Leics, UK). After 24h, amastigotes were added at a multiplicity of infection (MOI) of 10∶1 (in 300µl complete DMEM). After 1h at 37°C, non-adherent cells and free amastigotes were removed and cells were cultured for various times post infection (p.i.). In some instances, 2µm latex beads (Bangs Laboratories, Fishers, IN) were used in addition to or instead of amastigote infections. Cell lines were treated for 2h with IFN-α (1000U/ml; PBL Interferon Source, Piscataway, NJ) at the time of infection. To quantify infection level, cells were fixed and permeabilised using 4% paraformaldehyde + 0.1% Triton-X 100 (BDH) and coverslips were mounted in Vectashield with DAPI (Vector laboratories, Burlingame, CA). The proportion of cells infected and the number of amastigotes per 100 host cells was calculated from triplicate cultures per treatment group.

### Microarray analysis

10^6^ control or infected 14M1.4 and RAW264.7 cells were incubated for 12 and 48 hours in T25 tissue culture flasks. Samples were prepared in triplicate on three separate occasions, with an average infection level of 38±5% for 14M1.4 cells and 36±8% for RAW264.7 cells. RNA was extracted from the cell lines using Trizol according to the manufacturer's protocol (Sigma-Aldrich). One of the triplicate samples from each of the three experiments were used for microarray analysis. RNA concentration and integrity was established using 210 Bioanalyser (Agilent Technologies, Palo Alto, CA). Extracted RNA was reversed transcribed to cDNA using the Affymetrix GeneChip one-cycle target labelling kit (Affymetrix, Santa Clara, CA) according to the manufacturer's recommended protocols and hybridised to GeneChip® Mouse Genome 430 2.0 Genome Arrays. Raw data processing was performed by using the Affymetrix GCOS 1.2 software. After hybridization and scanning, probe cell intensities were calculated and summarized for the respective probe sets by means of the MAS5 algorithm. MAS5 normalised data were collected and analyzed by using the ArrayAssist Expression software, Version 5.5 (Stratagene). Differentially expressed genes were identified by using a two-class t test where significance level was set at p > 0.05. Genes that were >2.0 fold up- or down-regulated between groups were selected. Data from these studies have been deposited in the EBI ArrayExpress data base (Accession #: E-MEXP-2554). Gene lists were susbsequently analysed using Ingenuity Pathway Analysis (Ingenuity Systems, Redwood City, CA) and Expander (Shamir et al., 2005) software.

### Cell/parasite proliferation assay

Proliferation of macrophages and *L. donovani* amastigotes was assessed by analysis of the loss of CFSE (Invitrogen, Paisley, UK) fluorescence. Macrophages were incubated with 10µM CFSE in PBS for 10 minutes at 37°C, washed repeatedly, and then plated as above. At the appropriate times, macrophages were harvested and analysed for CFSE fluorescence. To assess amastigote proliferation, amastigotes labelled with CFSE as above prior to use for infection. At appropriate times p.i., macrophages were lysed with 1.25mg/ml saponin (BDH) in PBS. Released amastigotes were isolated by centrifugation and labelled with 10µM cell tracker blue (Invitrogen, Paisely, UK) for 20 minutes at 37°C, followed by specific anti-*L. donovani* antibody (1∶50 dilution of heat inactivated serum from an infected hamster) and then goat anti-hamster alexa fluor 647 antibody (10µg/ml; Invitrogen, Paisley, UK). CFSE dilution of macrophages and/or viable parasites was determined by flow cytometry using a cyan flow cytometer (Dako). 20,000–50,000 events were analysed per sample at each time point (n = 3).

### Real-time reverse transcription-PCR (RT-PCR)

Macrophage RNA was isolated using an RNeasy kit according to the manufacturer's instructions (Qiagen, UK). RNA was reverse transcribed to cDNA using the first-strand cDNA synthesis kit according to manufacturer's instructions (Invitrogen, Paisley, UK). Primers for the specific amplification of hypoxanthine phosphoribosyltransferase (HPRT) have been previously described ^(45)^. Pre-designed primers were used for the specific amplification of *Irf-7* and *Mx1* (PPM04696E and PPM05520A, respectively: Superarray, Frederick, MD). PCR conditions for all primers were 95°C (15s), 62°C (30s), and 60°C (30s) for 40 cycles. Real-time PCR was performed with the SYBR green PCR kit in an ABI Prism 7000 sequence detection system (Applied Biosystems) according to manufacturer's instructions. Relative expression of target genes was normalized to HPRT and was quantified by the formula 2-ΔΔCT where ΔΔCT  =  (CT Target − CT HPRT)_Treated_ − (CT Target − CT HPRT)_Control_. Values are expressed as the mean of triplicates ± SE. Broken line indicates the expression of the untreated control to which all samples were calibrated.

### IRF-7 flow cytometry

For IRF-7 protein expression, cells were fixed in 4% PFA and permeabilised on ice for 30 min with 0.1% Triton-X 100 buffer. Cells were incubated with a polyclonal rabbit anti-IRF-7 antibody (Zymed, San Francisco, CA) or control (rabbit IgG Calprologics Inc. Harwick, MA) used at 5µg/ml. Primary antibody was detected using a goat anti-rabbit alexa fluor 546 secondary antibody (Invitrogen, Paisley, UK). Flow cytometric analysis was performed with a CyAn flow cytometer and Summit v4.3 software (Dako).

### Fluorescence microscopy

Macrophages were stained for MyD88 and IRF-7 after fixation and permeabilised as above, but at room temperature. Cells were blocked in 2% goat serum (Vector laboratories) in 0.5% BSA (Fluka, UK) in PBS for 2h at room temperature or at 4°C overnight. Polyclonal rabbit anti-IRF-7 or control polyclonal sera was used at 5µg/ml. Monoclonal rat anti-MyD88 (R&D Systems, UK) or rat IgG2a (Invitrogen, Paisley, UK) was used at 5µg/ml. Primary antibodies were incubated with cells for 1h at RT. Primary antibodies were detected using the following secondary antibodies (10µg/ml; 1h at RT) ; goat anti-rabbit alexa fluor 546 (Invitrogen, Paisley, UK), and goat anti-rat alexa fluor 488 (Invitrogen, Paisley, UK). Coverslips were mounted in ProLong gold (Invitrogen, Paisley, UK) mounting medium. Immunofluorescence staining of 8µm cryosections of spleen was performed under same conditions except fixation/permeabilisation was carried out using ice cold acetone for 10 min. Sections were blocked as described above and antibodies were used as follows (5µg/ml); rat anti-CD169 FITC conjugated (Serotec, UK), rat IgG2a FITC isotype (Invitrogen), rat anti-SIGNR1 biotinylated (Bachem, UK), rat IgM biotinylated (Invitrogen). Streptavidin alexa fluor 633 (Invitrogen) was used to detect biotinylated primary antibodies and IRF-7/amastigote staining was carried out as above. Specificity of IRF-7 staining was determined by absence of staining in sections taken from naïve and infected B6.*Irf-7* ^−/−^ mice. Similarly, no specific staining around *L. donovani* amastigotes was observed following infection of 14M1.4 knock-down clones or following in vivo infection of B6.*Irf7* ^−/−^ mice (**Supplementary Figure S6**), confirming that the polyclonal anti-IRF-7 antibodies did not react to *Leishmania* components. Sections counterstained with 1µM DAPI (Invitrogen) in PBS for 10 minutes and mounted. Slides were analysed on a 4 laser (7-line) Zeiss Meta invert motorised microscope (Carl Zeiss, Germany) and images analysed in LSM software (Carl Zeiss, Germany). Total numbers of parasites within the MZ (and their distribution within macrophage subsets) were determined from >5 sections per mouse, scoring all MZ profiles observed in each section. Data were pooled across 3–5 mice per time point and each experiment was repeated independently at least twice.

### Statistical analysis

Statistical analysis was performed using two-tailed student t test or Mann Whitney U test (as appropriate), with *p* <0.05 considered significant.

## Supporting Information

Figure S1 CFSE dilution rates of host cells and *L. donovani* amastigotes. (A) 14M1.4 and RAW264.7 cells were labeled with CFSE and analysed for CFSE dilution by flow cytometry at various times thereafter. The average number of divisions per 24h was calculated over the entire 48h period and is shown in the histogram. (B) Amastigotes were labeled with CFSE prior to infection of 14M1.4 and RAW264.7 cells. At various times post infection, cells were lysed, amastigotes counterstained with anti-*L. donovani* and with cell tracker blue, and CFSE dilution analysed by flow cytometry. Histograms show percentage of amastigotes with CFSE dilution representing >1 or >2 divisions at each time point analysed.
(0.79 MB TIF)

Figure S2 Network maps of differentially expressed genes 12h after *L. donovani* infection. 14M1.4 and RAW264.7 cells were infected with *L. donovani* amastigotes and 12h later, RNA was extracted and used for gene profiling. Genes showing 2-fold up or down regulation were analysed using Ingenuity Pathways to identify networks. The 5 top scoring (for 14M1.4 cells) networks and the single network (for RAW264.7 cells) containing >20 differentially regulated genes are shown (A–E) 14M1.4 cells (F) RAW264.7.
(1.17 MB TIF)

Figure S3 Network maps of differentially expressed genes 48h after *L. donovani* infection. 14M1.4 and RAW264.7 cells were infected with *L. donovani* amastigotes and 48h later, RNA was extracted and used for gene profiling. Genes showing 2-fold up or down regulation were analysed using Ingenuity Pathways to identify networks. The 5 top scoring (for 14M1.4 cells) networks and the single network (for RAW264.7 cells) containing >20 differentially regulated genes are shown (A–E) 14M1.4 cells (F) RAW264.7.
(1.19 MB TIF)

Figure S4 Expression of IRF-7 in RAW 264.7 cells and bone marrow macrophages. (A) Real-time RT-PCR showing *Irf-7* mRNA accumulation in RAW264.7 cells following exposure to *L. donovani* at MOI of 30∶1 (diamond), 10∶1 (invert triangle), 1∶1 (triangle) or after exposure to 100µg/ml poly(I:C) (open square). (B) Expression of IRF-7 by confocal microscopy (red, IRF-7; blue, DAPI) in uninfected (left) and *L. donovani*-infected (centre) RAW264.7 cells, acquired and shown using the same setting as images shown in Figure 2. Right hand panel shows same image with IRF-7 intensity amplified to show lack of close association of IRF-7 and *Leishmania* phagosomes in RAW264.7 cells (c.f. Figure 3C and F). Scale bar: 10µm (C) 10-day CSF-1 bone marrow-derived macrophages without infection (left panel) and 6h after *L. donovani* infection (right panel) stained for IRF-7 (red) and counterstained with DAPI (blue). IRF-7 is not induced by infection and does not associate tightly with the *L. donovani* phagosome.
(1.25 MB TIF)

Figure S5 NO production by 14M1.4 cells. Nitric oxide production by 14M1.4 cells was determined in culture supernatants by Griess assay at the times indicated following infection with *L. donovani* amastigotes (6–48h) or at 24h after addition of 100µg/ml Poly (I,C) or IFNγ (100U/ml) plus LPS (10ng/ml). Data represent mean ± SEM from triplicate cultures.
(0.26 MB TIF)

Figure S6 Specificity of IRF-7 polyclonal antibody. (A, B) 14M1.4 cells (A) and knockdown line KD#1 (B) were infected with *L. donovani* and stained at 24h for IRF-7. (C,D) *L. donovani* infected B6 mice (C) and B6.*Irf7* ^−/−^ mice (D) were infected for 5h and then stained for IRF-7. No phagosomal staining of IRF-7 was observed in KD#1 or in B6.*Irf7* ^−/−^ mice, confirming that this antibody does not cross react with *Leishmania* components.
(4.89 MB TIF)

Table S1 Up- and down-regulated genes in 14M1.4 cells at 12h
(0.14 MB XLS)

Table S2 Up- and down-regulated genes in 14M1.4 cells at 48h
(0.12 MB XLS)

Table S3 Up- and down-regulated genes in RAW264.7 cells at 12h
(0.03 MB XLS)

Table S4 Up- and down-regulated genes in RAW264.7 cells at 48h
(0.03 MB XLS)

Video S1 IRF-7-MyD88 association in untreated 14M1.4 cells (red, IRF-7; green, MyD88; blue, DAPI.). For details, see text.
(2.10 MB MOV)

Video S2 Phagosomal ‘capping’ of IRF-7 in *Leishmania*-infected 14M1.4 cells (red, IRF-7; green, MyD88; blue, DAPI.). For details, see text.
(1.15 MB MOV)

Video S3 3D opacity projections of 14M1.4 macrophages infected for 24hrs with *L. donovani* amastigotes, and labelled with IRF-7 (red) and LAMP-1 (green) antibodies. Successive rotations show DAPI, IRF-7 and then LAMP-1. Note co-localisation of LAMP1 and IRF7 in phagosome left centre of image. For details, see text.
(9.81 MB MOV)

Video S4 MMM in *L. donovani* infected B6 mouse showing phagosomal localisation of IRF-7 (CD169^+^ green, IRF-7, red; LV9, white; DAPI, blue). For details, see text.
(1.77 MB MOV)
