# Supplementary figures and images for: Innate Killing of Leishmania donovani by Macrophages of the Splenic Marginal Zone Requires IRF-7
Source: PLoS Pathog. 2010 Mar 12;6(3):e1000813. doi: 10.1371/journal.ppat.1000813 (PMC2837405; doi:10.1371/journal.ppat.1000813)

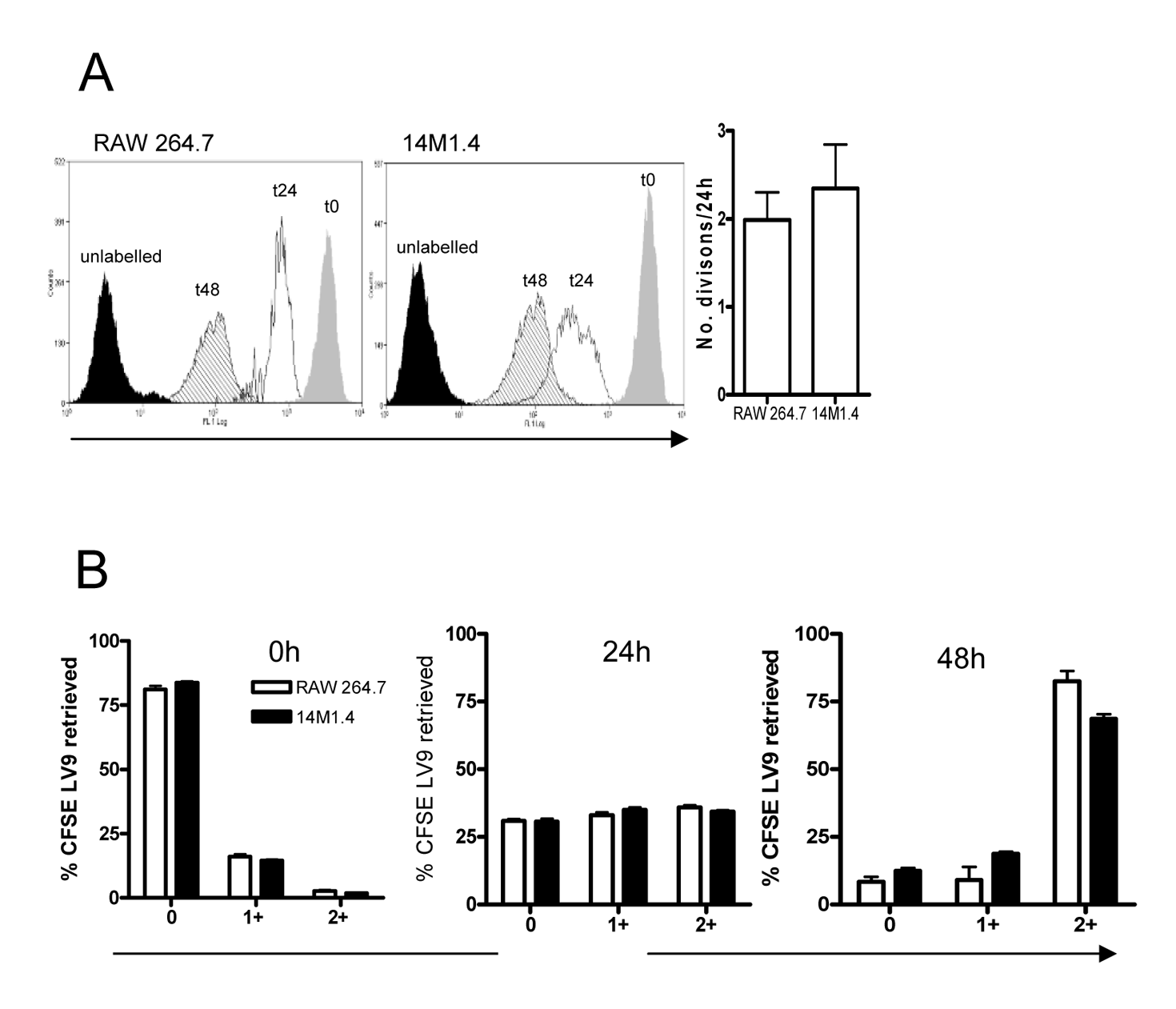

Supplement: Figure S1 — CFSE dilution rates of host cells and L. donovani amastigotes. (A) 14M1.4 and RAW264.7 cells were labeled with CFSE and analysed for CFSE dilution by flow cytometry at various times thereafter. The average number of divisions per 24h was calculated over the entire 48h period and is shown in the histogram. (B) Amastigotes were labeled with CFSE prior to infection of 14M1.4 and RAW264.7 cells. At various times post infection, cells were lysed, amastigotes counterstained with anti-L. donovani and with cell tracker blue, and CFSE dilution analysed by flow cytometry. Histograms show percentage of amastigotes with CFSE dilution representing >1 or >2 divisions at each time point analysed. (0.79 MB TIF) [file ppat.1000813.s001.tif]

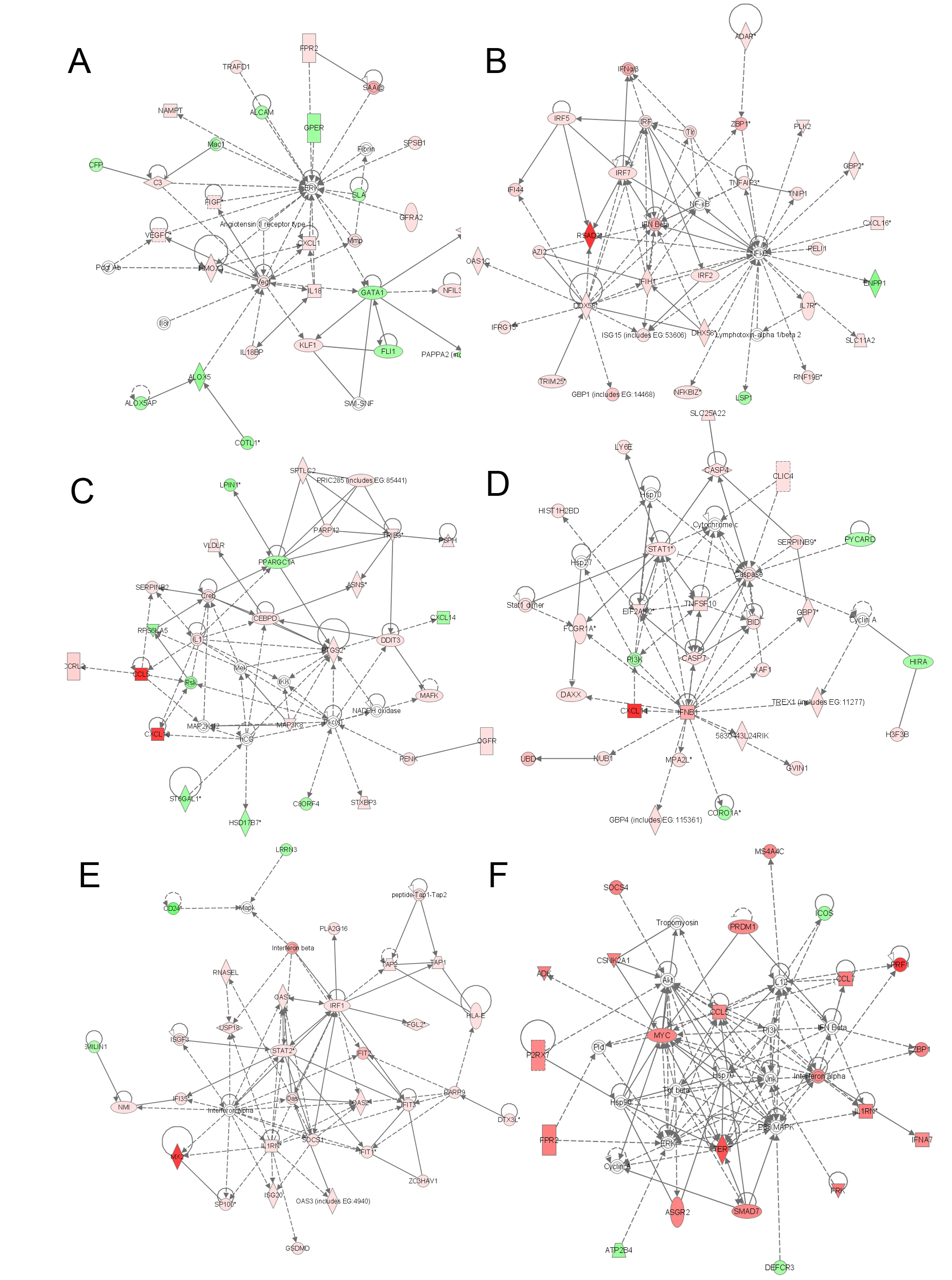

Supplement: Figure S2 — Network maps of differentially expressed genes 12h after L. donovani infection. 14M1.4 and RAW264.7 cells were infected with L. donovani amastigotes and 12h later, RNA was extracted and used for gene profiling. Genes showing 2-fold up or down regulation were analysed using Ingenuity Pathways to identify networks. The 5 top scoring (for 14M1.4 cells) networks and the single network (for RAW264.7 cells) containing >20 differentially regulated genes are shown (A–E) 14M1.4 cells (F) RAW264.7. (1.17 MB TIF) [file ppat.1000813.s002.tif]

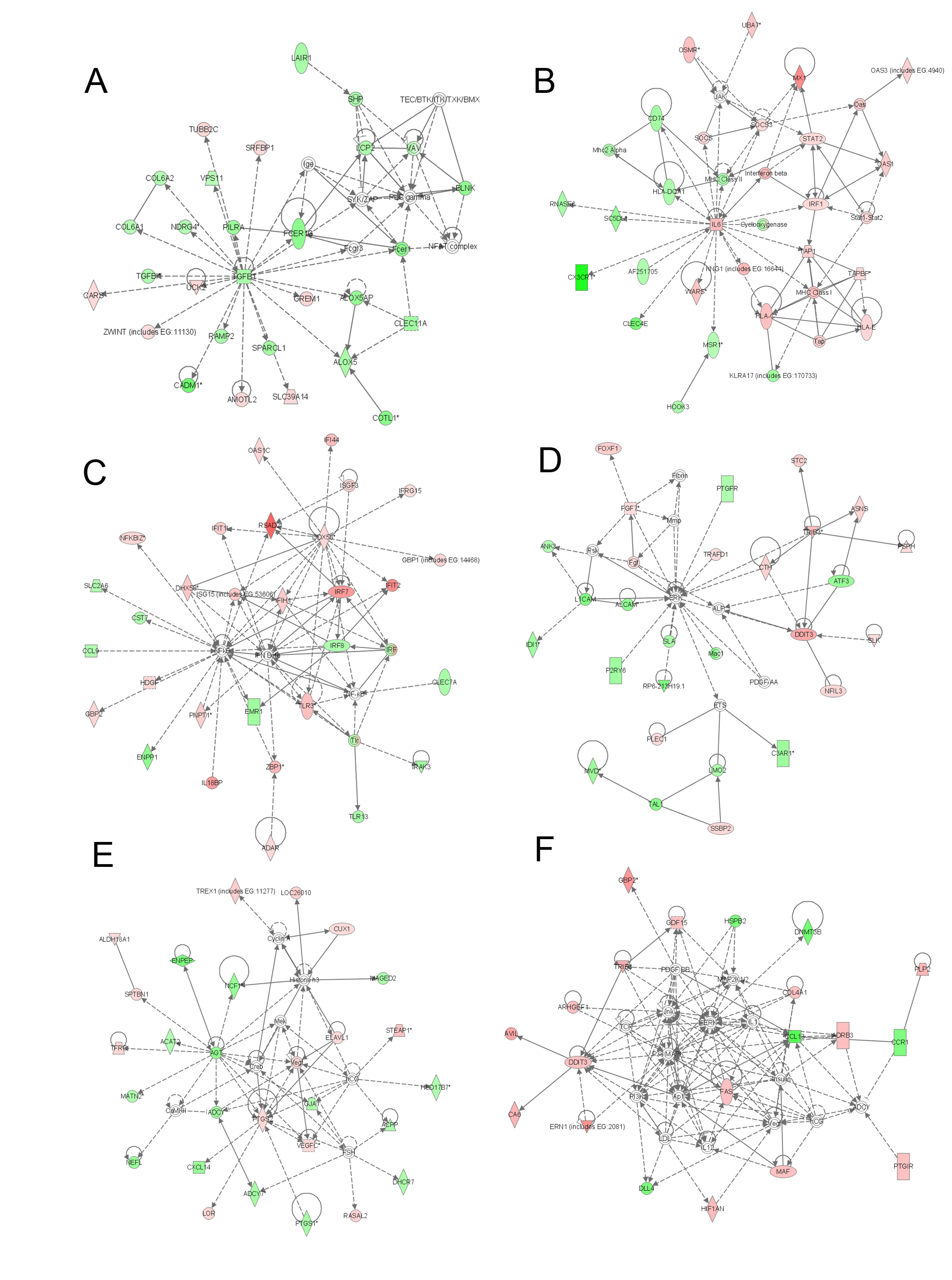

Supplement: Figure S3 — Network maps of differentially expressed genes 48h after L. donovani infection. 14M1.4 and RAW264.7 cells were infected with L. donovani amastigotes and 48h later, RNA was extracted and used for gene profiling. Genes showing 2-fold up or down regulation were analysed using Ingenuity Pathways to identify networks. The 5 top scoring (for 14M1.4 cells) networks and the single network (for RAW264.7 cells) containing >20 differentially regulated genes are shown (A–E) 14M1.4 cells (F) RAW264.7. (1.19 MB TIF) [file ppat.1000813.s003.tif]

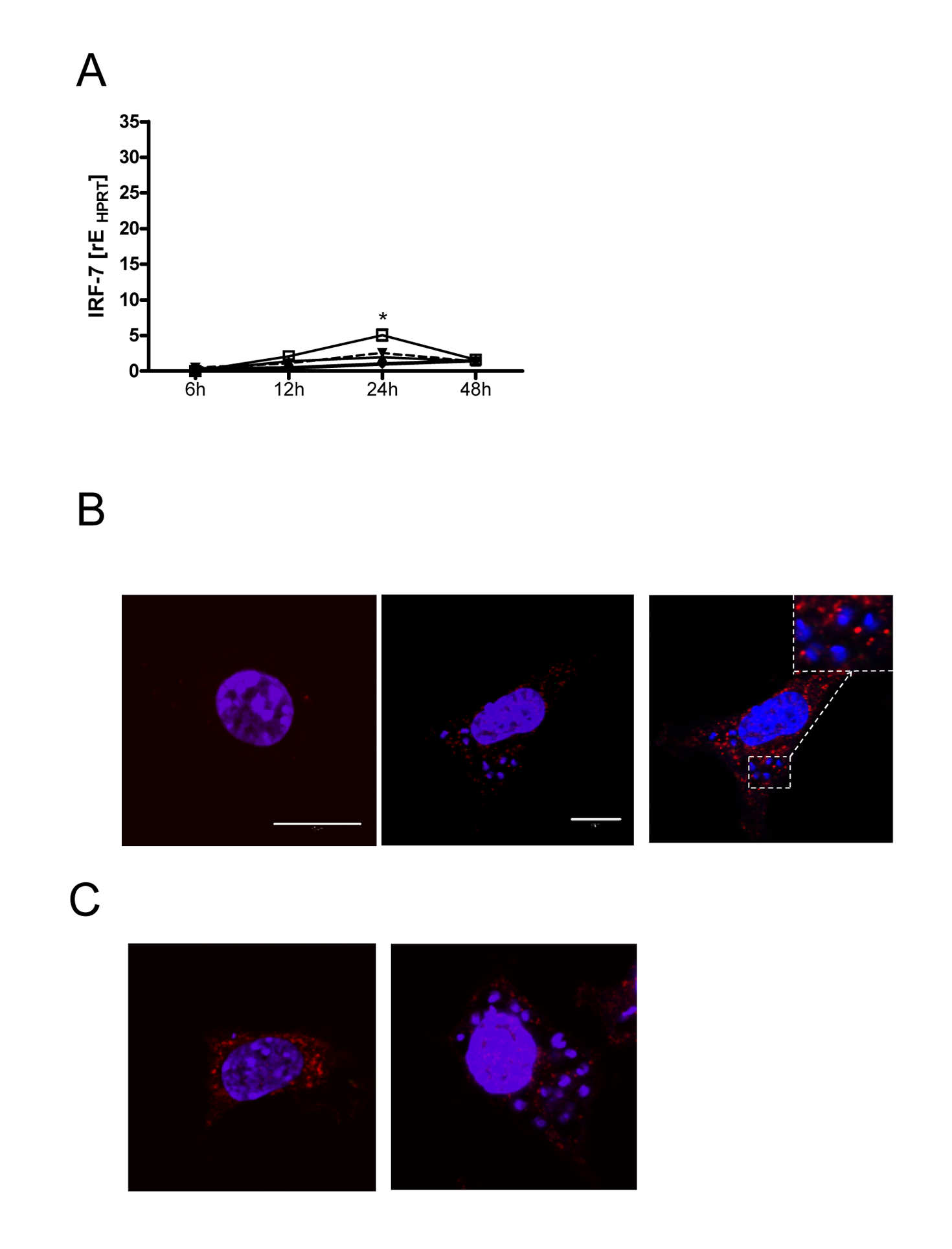

Supplement: Figure S4 — Expression of IRF-7 in RAW 264.7 cells and bone marrow macrophages. (A) Real-time RT-PCR showing Irf-7 mRNA accumulation in RAW264.7 cells following exposure to L. donovani at MOI of 30∶1 (diamond), 10∶1 (invert triangle), 1∶1 (triangle) or after exposure to 100µg/ml poly(I:C) (open square). (B) Expression of IRF-7 by confocal microscopy (red, IRF-7; blue, DAPI) in uninfected (left) and L. donovani-infected (centre) RAW264.7 cells, acquired and shown using the same setting as images shown in Figure 2. Right hand panel shows same image with IRF-7 intensity amplified to show lack of close association of IRF-7 and Leishmania phagosomes in RAW264.7 cells (c.f. Figure 3C and F). Scale bar: 10µm (C) 10-day CSF-1 bone marrow-derived macrophages without infection (left panel) and 6h after L. donovani infection (right panel) stained for IRF-7 (red) and counterstained with DAPI (blue). IRF-7 is not induced by infection and does not associate tightly with the L. donovani phagosome. (1.25 MB TIF) [file ppat.1000813.s004.tif]

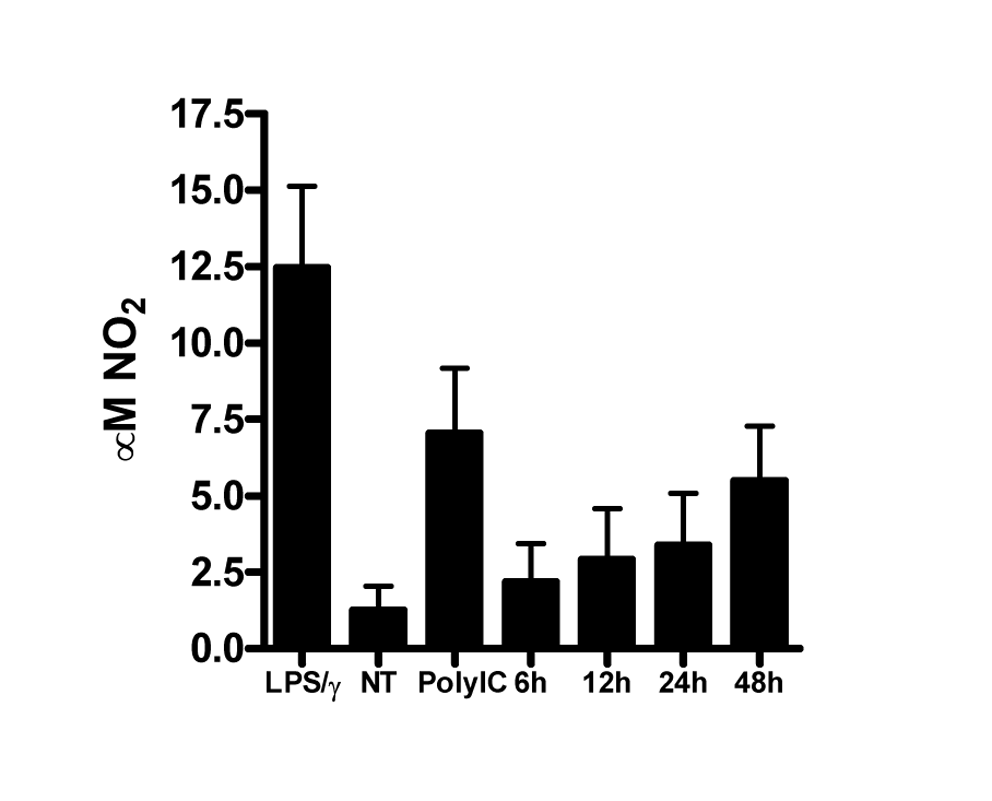

Supplement: Figure S5 — NO production by 14M1.4 cells. Nitric oxide production by 14M1.4 cells was determined in culture supernatants by Griess assay at the times indicated following infection with L. donovani amastigotes (6–48h) or at 24h after addition of 100µg/ml Poly (I,C) or IFNγ (100U/ml) plus LPS (10ng/ml). Data represent mean ± SEM from triplicate cultures. (0.26 MB TIF) [file ppat.1000813.s005.tif]

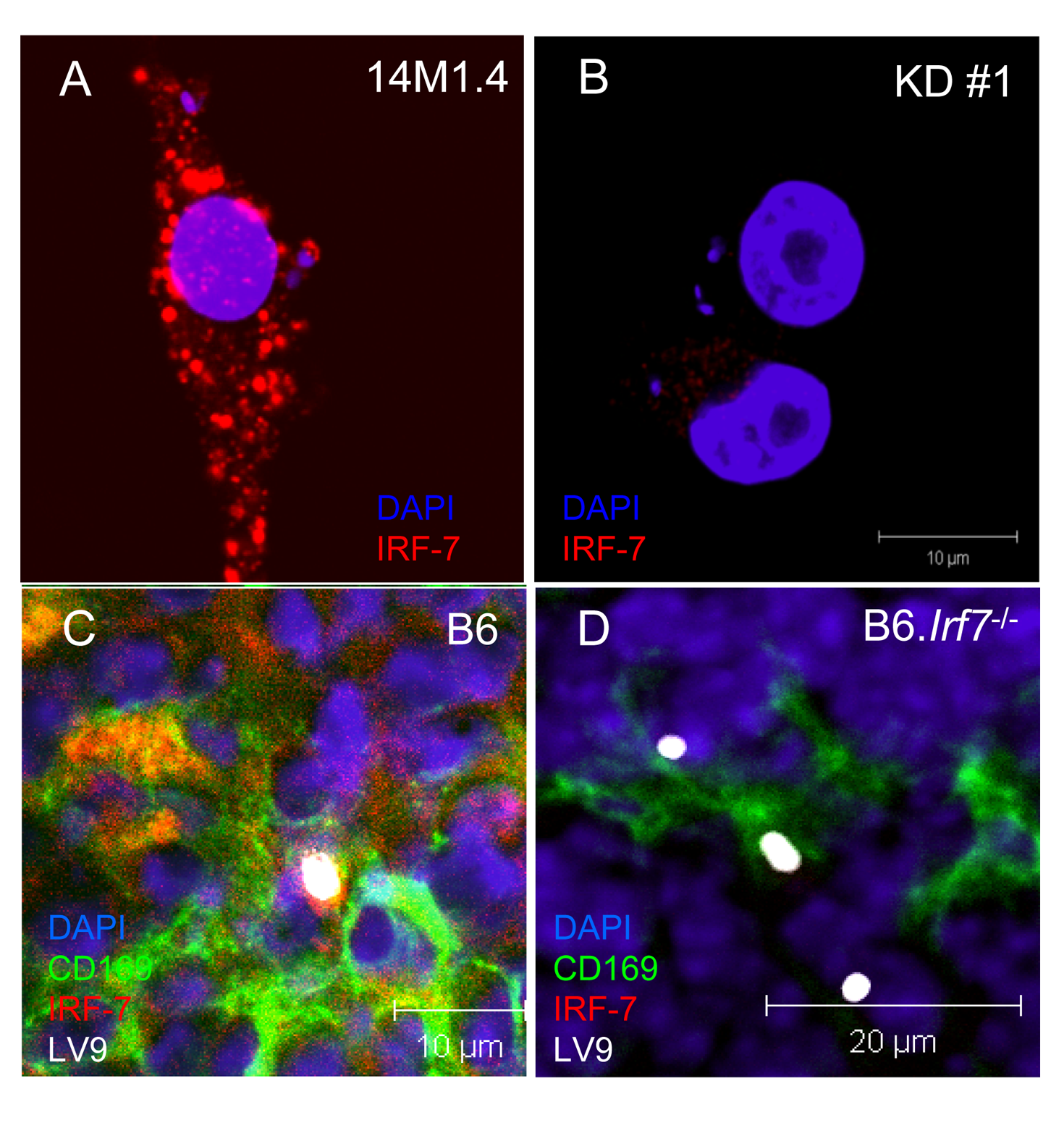

Supplement: Figure S6 — Specificity of IRF-7 polyclonal antibody. (A, B) 14M1.4 cells (A) and knockdown line KD#1 (B) were infected with L. donovani and stained at 24h for IRF-7. (C,D) L. donovani infected B6 mice (C) and B6.Irf7 −/− mice (D) were infected for 5h and then stained for IRF-7. No phagosomal staining of IRF-7 was observed in KD#1 or in B6.Irf7 −/− mice, confirming that this antibody does not cross react with Leishmania components. (4.89 MB TIF) [file ppat.1000813.s006.tif]
